# Hyperosmotic Stress Promotes the Nuclear Translocation of TFEB in Tubular Epithelial Cells Depending on Intracellular Ca^2+^ Signals via TRPML Channels

**DOI:** 10.1007/s12195-024-00839-6

**Published:** 2025-01-21

**Authors:** Takashi Miyano, Atsushi Suzuki, Hisaaki Konta, Naoya Sakamoto

**Affiliations:** 1 Department of Medical and Robotic Engineering Design, Tokyo University of Science, Tokyo, Japan; 2 Department of Mechanical Systems Engineering, Graduate School of Systems Design, Tokyo Metropolitan University, Tokyo, Japan

**Keywords:** Hyperosmolarity, Transcription factor EB (TFEB), Calcineurin, TRPML1, Tubular epithelial cell

## Abstract

**Purpose:**

We previously demonstrated that hyperosmotic stress, which acts as mechanical stress, induces autophagy of tubular epithelial cells. This study aims to elucidate the molecular mechanisms of hyperosmolarity-induced autophagy. The research question addresses how hyperosmotic stress activates autophagy through transcription factor EB (TFEB) and Ca^2+^ signaling pathways, contributing to understanding cellular responses to mechanical stress.

**Methods:**

NRK-52E normal rat kidney cells were subjected to hyperosmotic stress using mannitol-containing medium. Fluorescence microscopy was utilized to observe TFEB nuclear translocation, a crucial event in autophagy regulation. An intracellular Ca^2+^ chelator, BAPTA-AM, and a calcineurin inhibitor were used to dissect the Ca^2+^ signaling pathway involved in TFEB translocation. The phosphorylation of p70S6K, a substrate of the mammalian target of rapamycin complex 1 kinase, was analyzed to explore its role in TFEB localization. Additionally, the function of transient receptor potential mucolipin 1 (TRPML1), an intracellular Ca^2+^ channel, was assessed using pharmacological inhibition to determine its impact on TFEB translocation and autophagy marker LC3-II levels.

**Results:**

Mannitol-induced hyperosmotic stress promoted the nuclear translocation of TFEB, which was completely abolished by treatment with BAPTA-AM. Inhibition of calcineurin suppressed TFEB nuclear translocation under hyperosmolarity, indicating that a signaling pathway governed by intracellular Ca^2+^ is involved in TFEB’s nuclear translocation. In contrast, hyperosmotic stress did not significantly alter p70S6K phosphorylation. Pharmacological inhibition of TRPML1 attenuated both TFEB nuclear translocation and LC3-II upregulation in response to hyperosmotic stress.

**Conclusions:**

Hyperosmotic stress promotes TFEB nuclear localization, and TRPML1-induced activation of calcineurin is involved in the mechanism of hyperosmolarity-induced autophagy.

**Supplementary Information:**

The online version contains supplementary material available at 10.1007/s12195-024-00839-6.

## Introduction

Macroautophagy, referred to as autophagy hereafter, is a lysosome-mediated degradation and recycling process that regulates intracellular homeostasis [1]. Autophagy consists of multiple dynamic steps, including autophagosome formation, autolysosome formation, and degradation of cytoplasmic components, such as damaged organelles and misfolded proteins [2]. Autophagosomes, which are double-membrane vesicles, are tagged by a lipid-conjugated microtubule-associated protein 1 light chain 3 (LC3), commonly used as a autophagy marker. [3, 4]

The autophagy activity of tubular epithelial cells is indispensable for stress adaptation and homeostasis in the kidney [5–7]. Accumulating studies have demonstrated that several common renal insults, including ischemia, toxic injury, and inflammation, induce the activation of intracellular autophagy, particularly in the proximal tubular segments [8]. Proximal tubular epithelial cells are routinely exposed to severe changes in osmolarity, which are responsible for the reabsorption and secretion of various solutes. Our previous research revealed that sustained hyperosmotic mannitol stress, which acts as mechanical stress, could induce autophagy flux in proximal tubular epithelial cells [9]. Elucidating how hyperosmotic conditions induce autophagy will provide insights into the molecular pathways involved in mechanotransduction and responses of tubular cells to the conditions, which may have implications for various renal diseases.

Transcription factor EB (TFEB) is a major transcriptional regulator of autophagy-lysosome pathways that positively regulates the expression of autophagy and lysosomal genes [10, 11]. TFEB activity is significantly regulated by phosphorylation events [12]. While phosphorylated TFEB is retained in the cytoplasm and binds to 14-3-3 proteins, dephosphorylated TFEB travels to the nucleus to trigger the transcription of target genes. Phosphorylation of TFEB is mainly mediated by the mammalian target of rapamycin complex 1 (mTORC1) kinase, a major kinase complex that negatively regulates autophagy [13]. When mTORC1 activity is inhibited, TFEB is dephosphorylated and translocated into the nucleus [14]. In parallel, the activity of the calcium-dependent protein phosphatase calcineurin is induced by transient receptor potential mucolipin 1 (TRPML1), a lysosome-specific Ca^2+^-permeable ion channel, which contributes to the nuclear translocation of TFEB. [14–17] The overexpression of TRPML1 results in a significant increase in autophagic flux, whereas its deficiency can affect the accumulation of autophagosomes due to decreased autophagosome degradation and increased autophagosome formation. Thus, TRPML1 plays a crucial role in the early stages of autophagy. [18]

In the present study, we investigated the mechanisms underlying hyperosmolarity-induced autophagy by examining the effects of hyperosmotic stress on TFEB’s nuclear translocation and its mechanism in tubular epithelial cells. We demonstrated that the TRPML1 channel-induced activation of calcineurin caused TFEB nuclear translocation.

## Materials and Methods

### Cell Culture and Hyperosmotic Stimulation

NRK-52E (Japanese Collection of Research Bioresources, Osaka, Japan) normal rat tubular epithelial cells were grown in Dulbecco’s modified Eagle’s medium (DMEM, FUJIFILM Wako Pure Chemical, Osaka, Japan) supplemented with 10% heat-inactivated fetal bovine serum (Sigma-Aldrich, St. Louis, MO) and 1% penicillin/streptomycin (FUJIFILM Wako Pure Chemical). The cells were incubated at 37 °C under a humidified atmosphere with 5% CO_2_, and the culture medium was replaced every 3 to 4 days. NRK-52E cells were maintained in a complete medium to avoid starvation-induced autophagy. When the cells reached approximately 80% confluency, the medium was substituted with a hyperosmotic medium containing mannitol (FUJIFILM Wako Pure Chemical) or urea (FUJIFILM Wako Pure Chemical), as described previously [19]. For the inhibitor experiments, cells were pre-treated for 15 minutes with ethylene glycol tetraacetic acid (EGTA, 4 mM; FUJIFILM Wako Pure Chemical), 1,2-bis(2-aminophenoxy)ethane-N,N,N′,N′-tetraacetic acid (BAPTA-AM, 50 μM; Tokyo Kasei Kogyo, Tokyo, Japan), FK-506 (10 μM or 50 μM; Cayman Chemical, Ann Arbor, MI), ML-SI3 (1 μM or 10 μM; a TRPML antagonist, MedChemExpress, Monmouth Junction, NJ), or rapamycin (10 μM; FUJIFILM Wako Pure Chemical). All inhibitors were dissolved in dimethyl sulfoxide (DMSO) and diluted in culture medium to a final solvent concentration of 0.1%.

### Immunofluorescence Staining and Evaluation of Nuclear Translocation

All samples stained for immunofluorescence were imaged using an Olympus IX81 fluorescence microscope with a 60x objective. Images were captured at a resolution of 2048x2048 pixels with automatic exposure settings. After washing three times with phosphate-buffered saline (PBS), NRK-52E cells were fixed with 4% paraformaldehyde (FUJIFILM Wako Pure Chemical) at room temperature for 15 min. After treatment with membrane permeabilization solution (0.1% Triton X-100 in PBS) for 15 min, the cells were blocked with 3% bovine serum albumin (BSA, Sigma-Aldrich, Kyoto, Japan) in PBS for 1 h. The cells were then incubated with primary antibodies against rabbit anti-TFEB (1:200; Proteintech, Wuhan, China) or rabbit anti-nuclear factor of activated T cells (NFAT) (1:200; Invitrogen, Carlsbad, CA) in a blocking solution for 1 h, followed by incubation with a secondary antibody, anti-rabbit Ab conjugated to Alexa Fluor 488 or 546 (1:500; Invitrogen), for 1 h. Finally, the cells were incubated with Hoechst 33342 (Invitrogen) in the dark for 20 min to stain the nuclei.

To evaluate nuclear translocation, quantifications of fluorescence intensities in the nucleus and cytoplasm were performed using the ImageJ Fiji software (version 1.52b, NIH) [20]. Nuclear localization was estimated by dividing the average intensity of nuclear fluorescence by that of cytosolic fluorescence [21]. The nuclear region was determined based on the fluorescence emitted by Hoechst 33342, with the nuclear area extracted by applying an intensity threshold to isolate nuclei in each cell. The cytoplasmic region was determined by subtracting the nuclear region from the total cell area, which was identified through threshold-based extraction, with the outer cell boundary delineated using the TFEB fluorescent signal.

### RNA Extraction and Quantitative Real-Time PCR

Total RNA was isolated from NRK-52E cells using ISOGEN (NIPPON GENE, Toyama, Japan) and measured using a spectrophotometer (GE Healthcare, Piscataway, NJ). Subsequently, the total RNA was reverse-transcribed into complementary DNA using ReverTra Ace qPCR RT Master Mix (TOYOBO, Osaka, Japan). RT-qPCR was performed using THUNDERBIRD SYBR qPCR Mix (TOYOBO) and Thermal Cycler Dice Real-Time System Tp800 (TaKaRa Biomedicals, Shiga, Japan) according to the manufacturer’s instructions. PCR was conducted with 5 μM of cDNA, 10 μM of master mix, and 5 pM sense and antisense primers. The relative mRNA expression levels of the target genes in each sample were expressed as Ct values, which is the number of cycles required for the fluorescence signal to cross the defined threshold. The expression of each gene was normalized based on the expression of GAPDH, a housekeeping gene, and the relative gene expression levels of multiple experiments were calculated based on the 2^−ΔΔCt^ method. The primer sequences are shown in Table 1. The primers were designed to span exon-exon junctions to ensure specificity and prevent genomic DNA amplification. Additionally, melt curve analysis consistently displayed a single sharp peak for each target gene, confirming the specificity of the amplification.

**Table 1.** Primers used in this study

Gene Name	Sense	Antisense
Rat GAPDH	TGACAACTTTGGCATCGTGG	GGGCCATCCACAGTCTTCTG
Rat LC3	CCTGCTGCTGGCCGTAGT	TGATGAAGTCTTCCTGCCAAAA
Rat VPS18	GCTCCGCATTGACTTGGG	GCCTTCTGTCCATTGCGGT
Rat LAMP1	GCCCGCGTGACTCCTCTTCC	ACGCAGCAGTTCTTCTCCGT
Rat LAMP2	AGCAGGTGGTTTCCGTGTCTCG	AGGGCTGCTCCCACCGCTAT
Rat GAPDH	TGACAACTTTGGCATCGTGG	GGGCCATCCACAGTCTTCTG

### Western Blot

Protein samples were prepared using whole-cell lysates. After washing with PBS, NRK-52E cells were lysed in lysis buffer (150 mM NaCl, 1 mM EDTA, 50 mM Tris, 1% NP-40, and 1% Triton-X-100) containing 1% protease inhibitor cocktail (Sigma-Aldrich). Cell debris was removed by centrifugation at 10000 rpm for 10 min after sonication. Protein concentrations were determined using the DC Protein Assay kit (Bio-Rad, Hercules, CA) and an absorbance spectrophotometer (Bio-Rad). Protein samples were incubated with SDS sample buffer at 98 °C for 3 min. Equal amounts of protein (from 1 to 5 μg) were separated by SDS-polyacrylamide gel electrophoresis and transferred to polyvinylidene difluoride (PVDF) membranes. The PVDF membranes were blocked with 5% non-fat milk or 3% BSA in Tris-buffered saline containing 0.1% Tween 20 (TBST) for 1 h to prevent nonspecific binding. The membranes were incubated at 4 °C overnight with the following specific primary antibodies (all 1:2000): Anti-p70S6K (Cell Signaling Technology or CST, Danvers, MA), anti-phosphorylation p70S6K (Thr389; CST), and anti-LC3 (Abcam, Cambridge, MA), with anti- GAPDH (CST) serving as the internal control. Subsequently, the membranes were washed with TBST and incubated with horseradish peroxidase-linked anti-IgG secondary specific antibodies (1:2000; CST) at room temperature for 1 h. All blots were visualized using Pierce ECL Western Blotting Substrate (Thermo Fisher Scientific, Waltham, MA). The band intensities in the scanned membrane images were quantified using ImageJ.

To further investigate the subcellular localization and expression of TFEB and NFAT, NRK-52E cells were fractionated using a nuclear/cytosolic fractionation kit (Tokyo kasei, Tokyo, Japan) according to the manufacturer’s instructions. GAPDH and Lamin A/C (1:2000; CST) antibodies were used to detect internal controls in the cytoplasmic and nuclear fractions, respectively. The cytoplasmic and nuclear fractions were subsequently subjected to western blot analysis as described above.

### Data Analyses

All experiments were repeated at least three times. Summary data are presented as mean ± standard error (S.E.) of at least three separate experiments. In the parametric analysis, statistical significance was evaluated by Student’s *t*-test for two-group comparisons and by one-way analysis of variance (ANOVA) followed by Dunnett’s test for multiple-group comparisons. In the nonparametric analysis, statistical significance was evaluated by the Steel test for multiple-group comparisons. All statistical analyses were performed using the R 4.1.2 software (R Foundation for Statistical Computing, Vienna, Austria). A p-value < 0.05 was considered statistically significant. The number of data points is given by n in the figure legends.

## Results

### TFEB Localizes in the Nucleus in Response to the Mannitol-Mediated Hyperosmotic Stress

**Fig 1.**
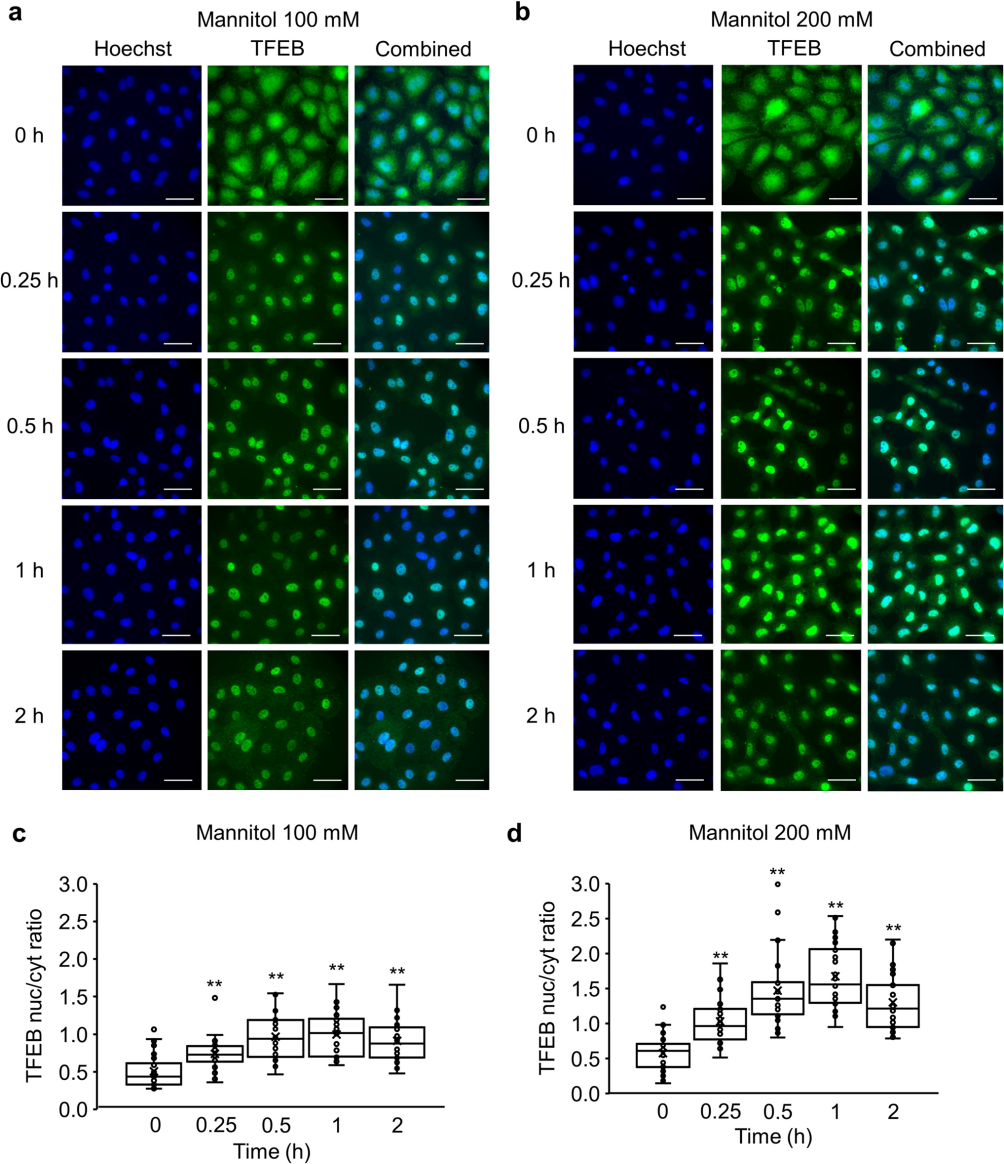
The effects of hyperosmotic stress mediated by mannitol on the nuclear translocation of TFEB in NRK-52E cells. (**a**, **b**) Representative fluorescence images of Hoechst 33342 (blue) and TFEB (green) of NRK-52E cells treated with 100 mM (**a**) or 200 mM (**b**) mannitol for 0, 0.25, 0.5, 1, and 2 h. Scale bar: 25 μm. (**c**, **d**) Summaries of the ratios between the nuclear and cytosolic TFEB fluorescence intensities in NRK-52E cells cultured with 100 mM (**c**) and 200 mM (**d**) mannitol. Data are presented as box and whisker plots with average (×), median, IQR, minimum value, and maximum value (100 mM: n = 30; 200 mM: n = 30). “n” represents the number of images analyzed. ^**^P < 0.01 vs. 0-h treatment (Dunnett’s test)

We first examined the effects of hyperosmotic stress on the subcellular localization of TFEB in the cytoplasm and nucleus by immunofluorescence staining (Fig. 1). In the absence of extracellular osmolytes (isotonic conditions, 0 h), the intracellular localization of TFEB was significantly higher in the cytoplasm than in the nucleus. When the cells were treated with 100 mM mannitol, TFEB translocated from the cytoplasm to the nucleus (Fig. 1a), with similar results observed following treatment with 200 mM mannitol (Fig. 1b). Quantitatively, after incubation of NRK-52E cells with 100 mM and 200 mM mannitol for all tested times (0.25, 0.5, 1, and 2 h), we observed a significant increase in the ratio of nuclear and cytoplasmic TFEB fluorescence intensities in a time-dependent manner, reaching a plateau at 1 h. 100 mM mannitol treatment yielded the following trend: 0 h (0.51 ± 0.04), 0.25 h (0.74 ± 0.04), 0.5 h (0.97 ± 0.05), 1 h (1.00 ± 0.06), and 2 h (0.91 ± 0.05). 200 mM mannitol treatment yielded the following trend: 0 h (0.57 ± 0.05), 0.25 h (1.03 ± 0.06), 0.5 h (1.47 ± 0.10), 1 h (1.67 ± 0.08), and 2 h (1.30 ± 0.07) (Fig. 1c,d). To confirm these findings, we performed Western blot analysis on nuclear and cytoplasmic fractions of cells treated with 100 and 200 mM mannitol. Consistent with the immunofluorescence data, the Western blot results showed an increase in the nuclear/cytoplasmic ratio of TFEB (Suppl. Fig 1).

**Fig 2.**
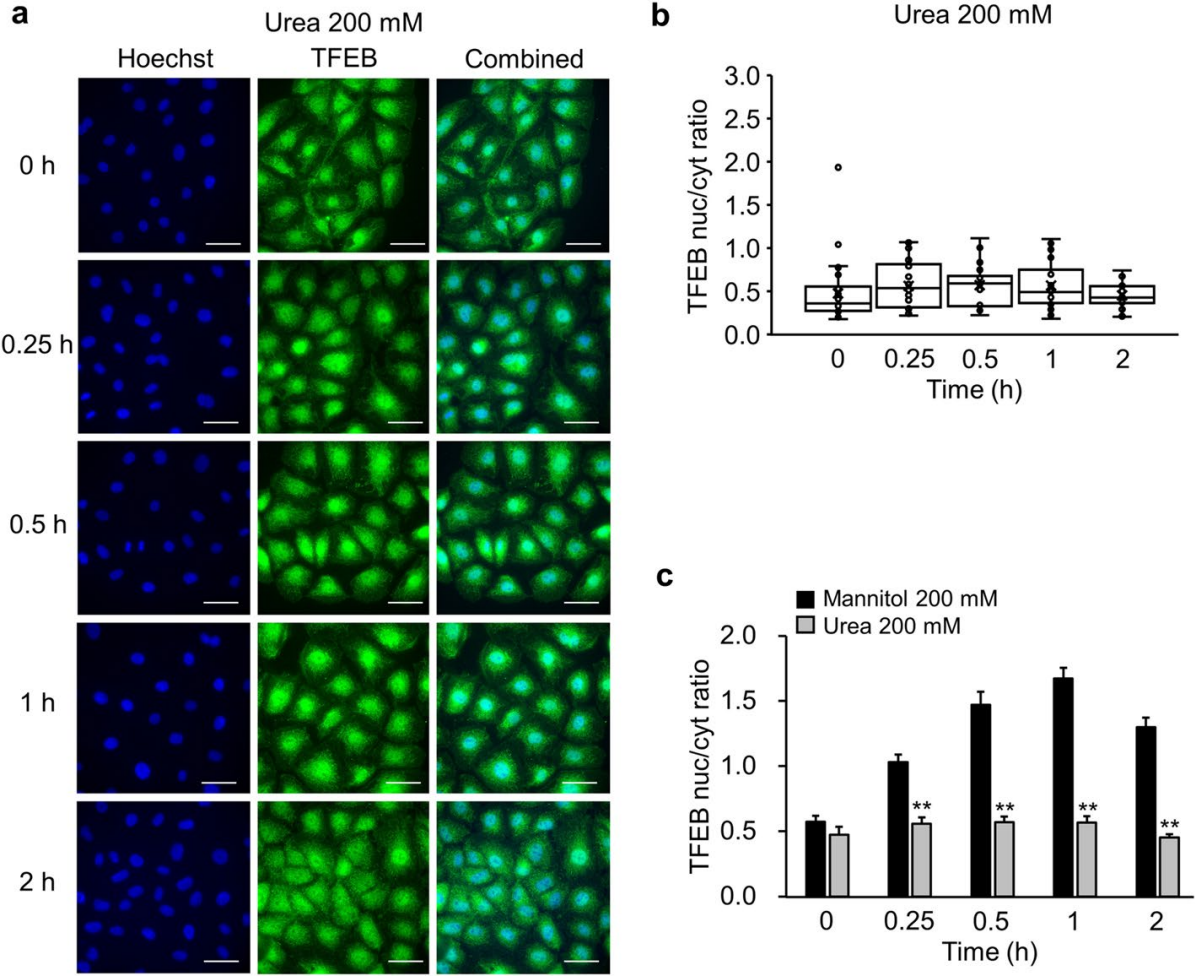
The effects of hyperosmotic stress mediated by urea on the nuclear translocation of TFEB in NRK-52E cells. **a** Typical fluorescence images of Hoechst 33342 (blue) and TFEB (green) of NRK-52E cells treated with 200 mM urea for 0, 0.25, 0.5, 1, and 2 h. Scale bar: 25 μm. **b** A summary of the ratio between the nuclear and cytosolic TFEB fluorescence intensities in NRK-52E cells cultured with 200 mM urea. Data are presented as box and whisker plots, with average (×), median, IQR, minimum value, and maximum values (n = 30). **c** A comparison between mannitol (200 mM) and urea (200 mM) from **b**. The data relating to the 200 mM mannitol group (Black bars) are identical to those in Fig. 1d and are shown for comparison. Data are presented as mean ± SEM. “n” represents the number of images analyzed. ^**^P < 0.01 vs. 200 mM mannitol treatment at the same time point (Student’s *t*-test)

We previously reported that mannitol and urea are osmolytes with different properties. Mannitol, a membrane-impermeable osmolarity regulator, can increase the expression of the autophagosome marker LC3 and enhance autophagy flux, as confirmed by using bafilomycin, whereas membrane-permeable urea cannot. [9] Thus, we confirmed the TFEB’s nuclear translocation in NRK-52E cells under urea-mediated hyperosmolarity to further understand the effects of hyperosmotic stress on autophagy. In contrast to the results obtained from treatments with mannitol, those from hyperosmotic urea-treatments yielded no significant change in the nuclear fluorescence intensity of TFEB compared to the control at all tested time points: 0 h (0.47 ± 0.06), 0.25 h (0.56 ± 0.05), 0.5 h (0.57 ± 0.04), 1 h (0.57 ± 0.05), and 2 h (0.45 ± 0.03) (Fig. 2a,b). At all tested time points, 200 mM urea treatment showed significantly lower ratio of nuclear and cytoplasmic TFEB fluorescence intensities compared to 200 mM mannitol treatment: 0.25 h (P < 0.01), 0.5 h (P < 0.01), 1 h (P < 0.01), and 2 h (P < 0.01) (Fig. 2c).

**Fig 3.**
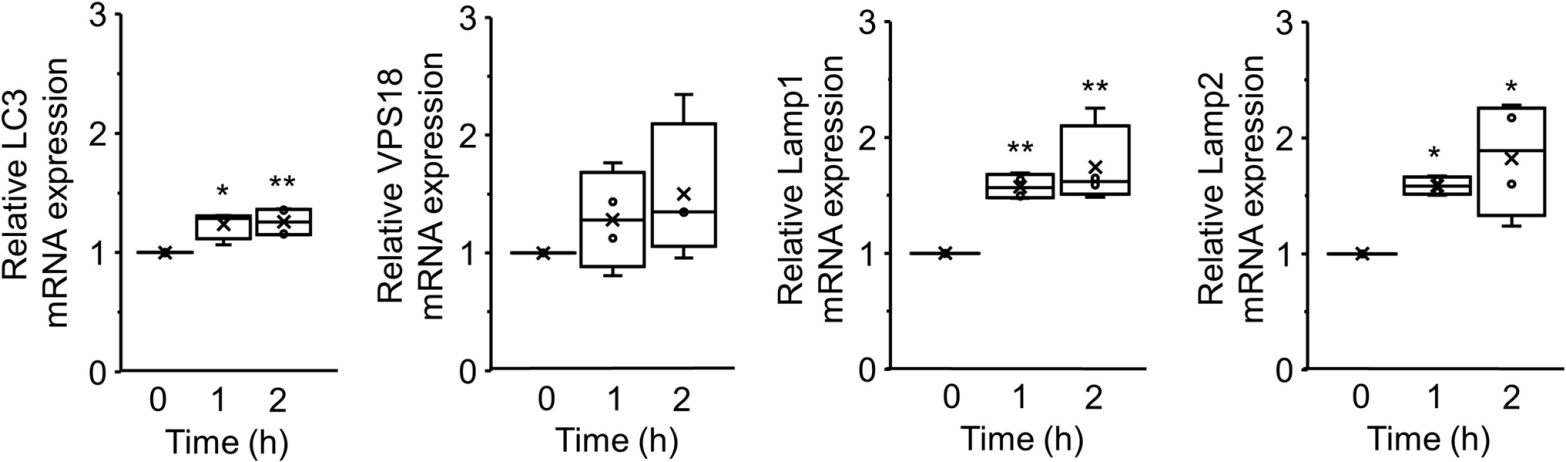
The effects of hyperosmotic stress mediated by mannitol on the transcriptional activity of TFEB. NRK-52E cells were treated with 200 mM mannitol, and their mRNA expression was analyzed by real-time PCR. Quantitation of the changes in LC3 (n = 4), VPS18 (n = 4), LAMP1 (n = 4), and LAMP2 (n = 4). Relative gene expression levels were calculated considering mannitol (0-h) as 1.0 and plotted. Data are presented as box and whisker plots, with average (×), median, IQR, minimum value, and maximum value. “n” represents the number of independent cultures. ^*^P < 0.05 and ^**^P < 0.01 vs. no treatment (Steel test)

Nuclear TFEB binds to the coordinated lysosomal expression and regulation element, which is found in the promoter regions of genes involved in lysosomal biogenesis and autophagy, to upregulate the expression of its target genes [10]. To further support the transactivation of TFEB by hyperosmotic mannitol stress, we measured the expression levels of the well-known target genes of TFEB, such as LC3, VPS18, LAMP1, and LAMP2. When the cells were stimulated by hyperosmotic mannitol stress, the relative mRNA expression levels of LC3 (1 h: 1.24 ± 0.06, P < 0.05; 2 h: 1.26 ± 0.06, P < 0.01), VPS18 (1 h: 1.28 ± 0.21; 2 h: 1.50 ± 0.30), LAMP1 (1 h: 1.57 ± 0.05, P < 0.01; 2 h: 1.74 ± 0.17, P < 0.01), and LAMP2 (1 h: 1.59 ± 0.04, P < 0.05; 2 h: 1.82 ± 0.25, P < 0.05) increased (Fig. 3).

Taken together, these results suggested that TFEB could be localized in the nucleus soon after the mannitol-mediated hyperosmotic stress was applied to the cells, resulting in the activation of its target genes.

### Hyperosmolarity-Induced Nuclear Translocation of TFEB Depends on the Activity of Calcineurin

**Fig 4.**
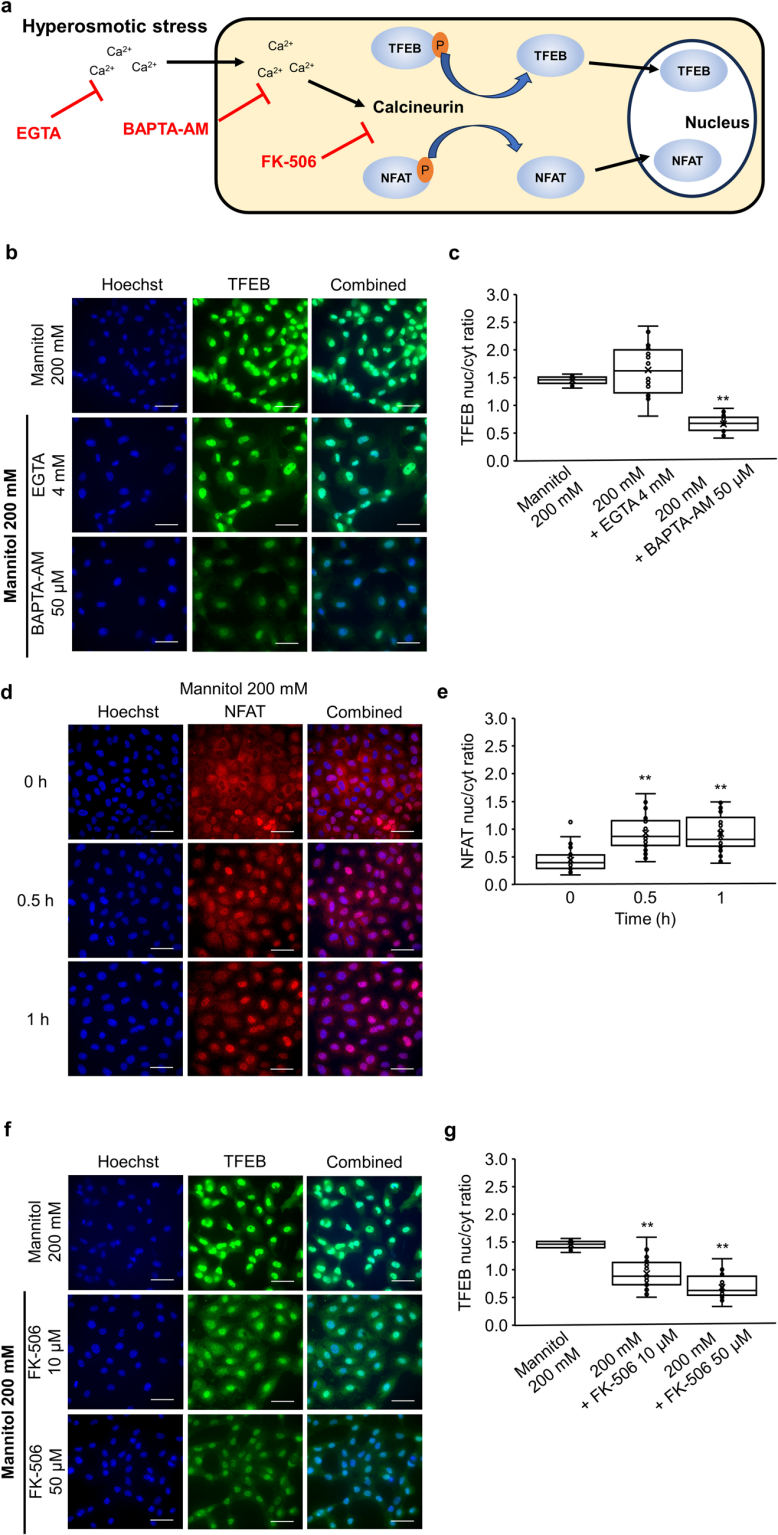
The effects of hyperosmotic stress mediated by mannitol on the calcineurin activity in NRK-52E cells. **a** A working model showing the role of Ca^2+^ in the hyperosmotic stress-induced TFEB’s nuclear translocation. Hyperosmotic stress causes an increase in the intracellular Ca^2+^ concentration, which activates calcineurin and leads to the dephosphorylation of TFEB and its subsequent nuclear translocation. **b** Representative fluorescence images of Hoechst 33342 (blue) and TFEB (green) staining in NRK-52E cells cotreated with mannitol (200 mM) and EGTA (4 mM) or BAPTA-AM (50 μM) for 1 h. Scale bar: 25 μm. **c** A summary of the ratios between the nuclear and cytosolic TFEB fluorescence intensities in NRK-52E cells. Data are presented as box and whisker plots, with average (×), median, IQR, and minimum and maximum values (200 mM mannitol + 4 mM EGTA: n = 20; 200 mM mannitol + 50 μM BAPTA-AM: n = 21). “n” represents the number of images analyzed. ^**^P < 0.01 vs. 200 mM mannitol treatment (Dunnett’s test). **d** Representative fluorescence images of Hoechst 33342 (blue) and NFAT (red) staining in NRK-52E cells treated with 200 mM mannitol for 0, 0.5, and 1 h. Scale bar: 25 μm. **e** A summary of the ratios between the nuclear and cytosolic NFAT fluorescence intensities in NRK-52E cells cultured with 200 mM mannitol. ^**^P < 0.01 vs. no treatment (Dunnett’s test). Data are presented as box and whisker plots, with average (×), median, IQR, minimum value, and maximum value (n = 30). “n” represents the number of images analyzed. **f** Representative fluorescence images of Hoechst 33342 (blue) and TFEB (green) staining in NRK-52E cells cotreated with mannitol (200 mM) and FK-506 (10 μM or 50 μM) for 1 h. Scale bar: 25 μm. **g** A summary of the ratios between the nuclear and cytosolic TFEB fluorescence intensities in NRK-52E cells. The data relating to the 200 mM mannitol alone group are identical to those in Fig. 4c (at 1 h) and are shown for comparison. Data are presented as box and whisker plots, with average (×), median, IQR, minimum value, and maximum value (200 mM mannitol + 10 μM FK-506: n = 24; 200 mM mannitol + 50 μM FK-506: n = 20). “n” represents the number of images analyzed. ^**^P < 0.01 vs. 200 mM mannitol treatment (Dunnett’s test)

Ca^2+^ is the primary regulator of autophagy and TFEB’s nuclear translocation [22], and we previously reported that hyperosmotic stress increases intracellular Ca^2+^ concentration in NRK-52E cells [23]. Based on the results of previous studies, we developed a working model that illustrates the role of Ca^2+^ in the hyperosmotic stress-induced TFEB nuclear translocation (Fig. 4a). To clarify the role of Ca^2+^ during this process, NRK-52E cells were treated with Ca^2+^ chelators before mannitol stimulation. As a result, treatment with a specific intracellular Ca^2+^ chelator, BAPTA-AM (50 μM), strongly reduced the hyperosmotic stress-induced TFEB nuclear translocation (0.66 ± 0.03, P < 0.01), but treatment with an extracellular Ca^2+^ chelator, EGTA (4 mM), showed no significant effect compared to 200 mM mannitol treatment, yielding 1.64 ± 0.10 and 1.67 ± 0.08, respectively (Fig. 4b,c). These results indicated that intracellular Ca^2+^ is required for hyperosmolarity-induced TFEB activation.

Because the Ca^2+^-dependent dephosphorylation of TFEB by calcineurin is known to be the primary mechanism of TFEB’s nuclear translocation [24], we next investigated whether hyperosmotic stress modulates the activity of calcineurin. This was evaluated by the nuclear localization of the transcription factor NFAT, which is translocated from the cytoplasm into the nucleus on calcineurin-dependent dephosphorylation [25]. Immunofluorescence staining showed that treatment with 200 mM mannitol promoted NFAT’s translocation from the cytoplasm to the nucleus (Fig. 4d). Quantitatively, after incubating NRK-52E cells with 200 mM mannitol for 0.5 or 1 h, we observed a significant increase in the ratio of nuclear and cytoplasmic NFAT fluorescence intensities (0 h: 0.44 ± 0.04 vs. 0.5 h: 0.92 ± 0.06, P < 0.01; 1 h: 0.92 ± 0.06, P < 0.01) (Fig. 4e). To confirm these findings, we conducted Western blot analysis of nuclear and cytoplasmic fractions from cells treated with 200 mM mannitol at the 0.5 and 1 h time points. The Western blot results confirmed the immunofluorescence findings, showing a significant increase in the nuclear/cytoplasmic ratio of NFAT, consistent across both time points (Suppl. Fig 2a,b). To further determine whether the calcineurin signaling pathway modulates hyperosmotic stress-induced TFEB activation, we assessed the pharmacological impact of FK-506, a calcineurin-specific inhibitor. FK-506 exerts its effect by binding to the FK506-binding protein (FKBP12), thereby inhibiting calcineurin enzymatic activity and preventing the dephosphorylation of NFAT, which, in turn, blocks its nuclear translocation. To confirm these effects, we performed Western blot analyses of nuclear and cytoplasmic fractions. The results showed a significant decrease in the nuclear/cytoplasmic ratio of NFAT following FK-506 treatment in cells exposed to 200 mM mannitol (Suppl. Fig 2c, d), providing further evidence that FK-506 inhibits calcineurin activity. Cotreatment with FK-506 effectively inhibited the hyperosmotic stress-induced TFEB nuclear translocation in a dose-dependent manner (200 mM mannitol: 1.67 ± 0.08 vs. 10 μM FK-506: 0.93 ± 0.06, P < 0.01; 50 μM FK-506, 0.69 ± 0.05, P < 0.01) (Fig. 4f,g). These findings suggested that the activation of calcineurin by hyperosmotic stress is involved in the nuclear translocation of TFEB.

**Fig 5.**
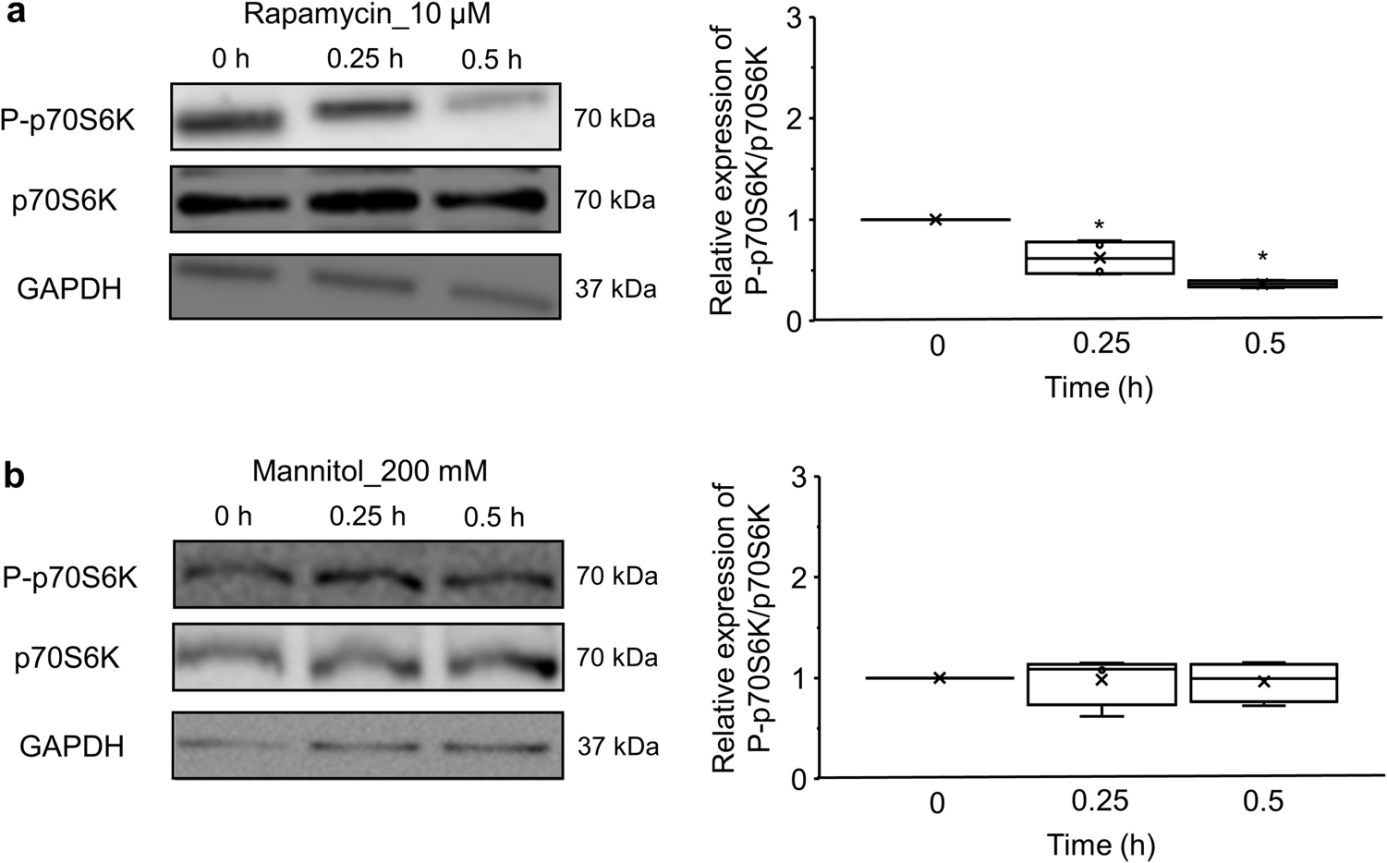
The effects of hyperosmotic stress mediated by mannitol on the protein expression of P‑p70S6K and p70S6K. **a**, **b** Western blot analysis for P‑p70S6K and p70S6K in NRK-52E cells treated with 10 μM rapamycin **a** or 200 mM mannitol **b** for 0, 0.25, and 0.5 h. Left: Representative western blot images. GAPDH served as the loading control. Right: Quantification analysis of the ratio of P‑p70S6K to p70S6K. The expressions of P‑p70S6K and p70S6K were normalized to that of GAPDH. Relative expression of P‑p70S6K/p70S6K was calculated by normalizing to the expression level at 0 h, or with no treatment. Data are presented as box and whisker plots, with average (×), median, IQR, minimum value, and maximum value (10 μM rapamycin: n = 4; 200 mM mannitol: n = 4). “n” represents the number of independent cultures. ^*^P < 0.05 vs. 0-h treatment (Steel test)

Because the localization of TFEB is not only regulated by phosphatase calcineurin but also by mTORC1, we further evaluated the effects of hyperosmotic treatment on the mTORC1 substrate, p70S6 kinase (p70S6K), whose phosphorylation levels (P-p70S6K) reflect mTORC1 activity (Fig. 5) [26]. Western blot analysis showed that treatment with rapamycin, mTORC1 inhibitors, significantly attenuated the expression ratio of P-p70S6K to p70S6K (0.25 h: 0.62 ± 0.09, P < 0.05; 0.5 h: 0.37 ± 0.02, P < 0.05) (Fig. 5a). On the contrary, 200 mM mannitol treatment did not significantly alter the expression ratio of P-p70S6K to p70S6K (0.25 h: 0.99 ± 0.12; 0.5 h: 0.97 ± 0.10) (Fig. 5b). These results suggested that hyperosmotic stress can promote TFEB’s nuclear translocation in NRK-52E cells in the mTORC1-independent manner.

### Cotreatment with ML-SI3 Inhibits the Hyperosmolarity-Induced TFEB Nuclear Translocation

The TRPML1 channel is predominantly localized in the membranes of late endosomes and lysosomes, including NRK cells [27]. Previous studies have reported that TRPML1 plays an important role in the activation of calcineurin and consequent TFEB dephosphorylation [18, 24, 28]. Therefore, we sought to investigate the role of TRPML1 channels in the hyperosmotic mannitol-induced TFEB nuclear translocation in NRK-52E cells.

**Fig 6.**
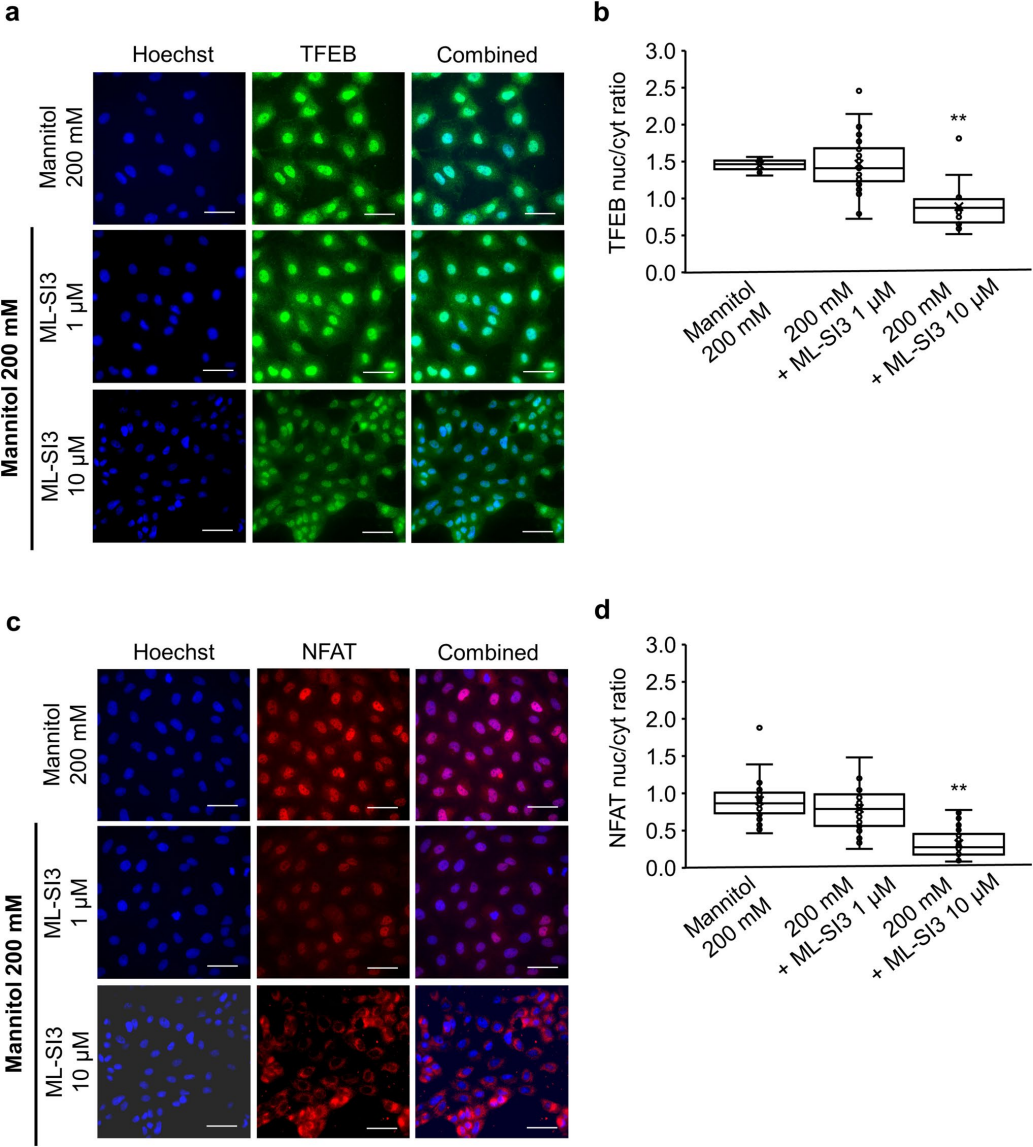
The effects of ML-SI3 on the mannitol-mediated hyperosmotic stress-induced nuclear translocation of TFEB in NRK-52E cells. **a** Representative fluorescence images of Hoechst 33342 (blue) and TFEB (green) staining of NRK-52E cells cotreated with mannitol (200 mM) and ML-SI3 (1 μM or 10 μM) for 1 h. Scale bar: 25 μm. **b** A summary of the ratios between the nuclear and cytosolic TFEB fluorescence intensities of NRK-52E cells. The data relating to the 200 mM mannitol alone group are identical to those in Fig. 4c (at 1 h) and are shown for comparison. Data are presented as box and whisker plots, with average (×), median, IQR, minimum value, and maximum value (200 mM mannitol + 1 μM ML-SI3: n = 29; 200 mM mannitol + 10 μM ML-SI3: n = 20). “n” represents the number of images analyzed. ^**^P < 0.01 vs. 200 mM mannitol treatment (Dunnett’s test). **c** Representative fluorescence images of Hoechst 33342 (blue) and NFAT (red) staining of NRK-52E cells cotreated with mannitol (200 mM) and ML-SI3 (1 μM or 10 μM) for 1 h. Scale bar: 25 μm. **d** A summary of the ratios between the nuclear and cytosolic NFAT fluorescence intensities of NRK-52E cells. Data are presented as box and whisker plots, with average (×), median, IQR, minimum value, and maximum value (200 mM mannitol + 1 μM ML-SI3: n = 28; 200 mM mannitol + 10 μM ML-SI3: n = 30). “n” represents the number of images analyzed. ^**^P < 0.01 vs. 200 mM mannitol treatment (Dunnett’s test)

Treatment with ML-SI3, a TRPML1 antagonist, attenuated the nuclear localization of TFEB in response to 200 mM mannitol treatment, with the most significant effects observed at high doses (1 μM ML-SI3: 1.47 ± 0.07 vs. 10 μM ML-SI3: 0.89 ± 0.06, P < 0.01) (Fig. 6a,b). Western blot analysis revealed that overall TFEB protein expression levels remained unchanged across all treatment groups (Suppl. Fig 3). Similar results were observed for the nuclear translocation of NFAT (1 μM ML-SI3: 0.80 ± 0.06 vs. 10 μM ML-SI3: 0.32 ± 0.04, P < 0.01) (Fig. 6c,d).

**Fig 7.**
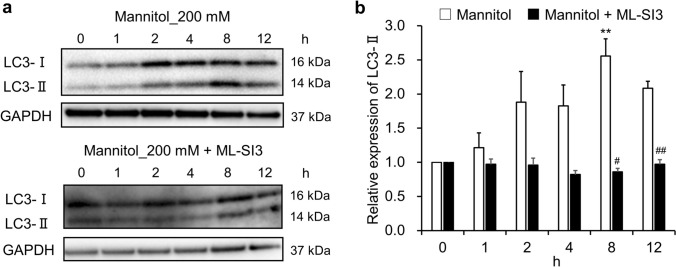
The effects of ML-SI3 on the mannitol-mediated hyperosmotic stress-induced LC3-II upregulation in NRK-52E cells. **a**, **b** NRK-52E cells were treated with 200 mM mannitol or cotreated with ML-SI3 (10 μM) for 0, 1, 2, 4, 8, and 12 h. **a** Representative blot images of LC3-I and LC3-II in NRK-52E cells treated with 200 mM mannitol alone (top) or cotreated with 10 μM ML-SI3 (bottom), where GAPDH served as the loading control. **b** Relative LC3-II expression level was calculated by normalizing to the level at 0 h, or with no treatment, and plotted as mean ± SEM (mannitol: n = 3; mannitol + ML-SI3: n = 4). “n” represents the number of independent cultures. ^**^P < 0.01 vs. 0-h or no treatment (Steel test). ^#^P < 0.05 and ^##^P < 0.01 vs. 200 mM mannitol treatment (Student’s *t*-test)

Moreover, to observe the effects of TRPML1 on autophagy in response to hyperosmotic stress, NRK-52E cells were cotreated with ML-SI3 and mannitol, before conducting a western blot analysis on the protein level of LC3-II. LC3 exists in two forms: LC3-I, a cytosolic form, and LC3-II, a lipidated form associated with autophagosome membranes. The conversion of LC3-I to LC3-II is a well-established marker of autophagy initiation and autophagosome formation. Stimulation of cells with 200 mM mannitol led to a significant increase in LC3-II expression in a time-dependent manner (1 h: 1.21 ± 0.22; 2 h: 1.88 ± 0.45; 4 h: 1.83 ± 0.31; 8 h: 2.56 ± 0.25, P < 0.01; 12 h: 2.09 ± 0.10) (Fig. 7a,b). Even under hyperosmotic conditions, treatment with ML-SI3 attenuated the increase in LC3-II expression to a level comparable to that of the control (1 h: 0.97 ± 0.08; 2 h: 0.96 ± 0.10; 4 h: 0.82 ± 0.06; 8 h: 0.86 ± 0.05, P < 0.05 vs. 200 mM mannitol treatment; 12 h: 0.97 ± 0.07, P < 0.01 vs. 200 mM mannitol treatment) (Fig. 7a,b). These findings indicated that the TRPML1-mediated nuclear translocation of TFEB plays an important role in hyperosmotic stress-induced autophagy in NRK-52E cells.

## Discussion

The primary purpose of this study was to explore the mechanisms of hyperosmolarity-induced autophagy of proximal tubular epithelial cells. The novel finding of this study is that mannitol-mediated hyperosmotic stress can promote TFEB nuclear localization and that TRPML1-induced activation of calcineurin is likely involved in the mechanism of hyperosmotic stress-induced autophagy. TRPML1, a lysosomal calcium channel, has been shown to mediate the release of Ca^2^⁺ from lysosomes in response to various stimuli. This calcium release can activate calcineurin, a calcium-dependent phosphatase, which subsequently dephosphorylates TFEB, thereby promoting its translocation into the nucleus and initiating autophagy.

Mannitol and urea have different properties; mannitol is a membrane-impermeable osmolyte that can induce cell shrinkage under hyperosmotic conditions, whereas urea permeates through the cell membrane and does not induce cell shrinkage [9]. As shown in Fig 1, the nuclear translocation of TFEB was observed following the hyperosmotic stress mediated by mannitol, but not under the stress mediated by urea. Considering that TFEB is the master regulator of autophagy, these results are consistent with our previous findings that hyperosmotic mannitol treatment induces autophagy flux, but urea treatment does not [9]. Thus, the mannitol-induced TFEB nuclear localization and the subsequent induction of autophagy could be triggered by a mechanically driven process resulting from cell shrinkage due to osmotic differences between the cytosol and the extracellular compartment and not by the hyperosmotic condition itself. However, we acknowledge that hyperosmotic stress also affects intracellular ion concentrations, including Ca^2+^ levels, which play a critical role in signaling pathways such as calcineurin activation. While urea, which does not induce compression, did not show the same effects as mannitol in our experiments, it remains uncertain whether urea similarly influences Ca^2+^ concentration. Furthermore, although we observed a trend toward increased VPS18 expression in response to hyperosmotic stress, this increase was not statistically significant. One possibility is that the hyperosmotic stress-induced activation of TFEB specifically upregulates genes directly involved in autophagy and lysosomal function, such as LC3, LAMP1, and LAMP2, which showed significant increases. The cellular responses to osmotic stress, including cell shrinkage and ion concentration changes, may trigger unique pathways or modify the timing and extent of autophagy induction. Further investigation will be needed to elucidate these potential differences in the underlying mechanisms.

Although basal autophagic activity in the proximal tubule is higher in the aged kidney than in the young kidney, autophagic flux in response to stress is blunted with aging in a transgenic mouse expressing a green fluorescent protein-LC3, which may be associated with age-related kidney disease [7, 29]. In addition, autophagy deletion in the proximal tubules worsens tubular injury and renal functions, indicating that enhanced autophagic activities may have renoprotective effects in various pathological models [30]. Furthermore, both mRNA and protein expression levels of TFEB are decreased in the kidneys of patients with diabetic kidney disease, and the dysfunction of the autophagy-lysosome pathway results in the accumulation of misfolded proteins and damaged organelles [31]. Thus, pharmacological approaches to modulate autophagy focused on TFEB hold promise for treating kidney diseases. The mTORC1 inhibitors represent a class of drug candidates that enhance autophagic activities. Indeed, previous studies have shown that abnormal mTORC1 hyperactivation, which leads to TFEB inactivation, is involved in the pathogenesis of tubular damage in diabetic kidney disease [32]. However, a meta-analysis of randomized clinical trials revealed that the relative risk of all grades of acute kidney injury in patients taking mTOR inhibitors is significantly higher than that in patients not taking mTOR inhibitors, indicating that renal toxicity is a potential complication of mTOR inhibitor use [33]. Interestingly, hyperosmotic stress was largely dispensable for the phosphorylation of the mTORC1 substrate p70S6K (Fig. 5b), which behaved differently from rapamycin, a typical mTOR inhibitor (Fig. 5a). Although a systematic analysis of the other downstream targets, such as eukaryotic initiation factor 4E-binding protein 1 (4E-BP1), can lead to a more comprehensive understanding, our results suggest that hyperosmotic stress promotes TFEB nuclear translocation by a different mechanism of rapamycin. Recent studies have shown that TRPML1 activation induces TFEB nuclear localization in a manner dependent on mTORC1 suppression, but not downregulation of p70S6K phosphorylation [34, 35]. To further elucidate the mechanism of hyperosmotic stress-induced TFEB nuclear translocation, it is important to examine the effects on the phosphorylation of Serine 211 of TFEB, a direct target of mTORC1. [24]

There are mTORC1-independent pathways that can also induce autophagy. The activation of calcineurin by hyperosmotic stress may lead to TFEB activation (Fig. 3), as calcineurin is known to enhance the nuclear translocation of TFEB. Several positive effects of calcineurin activation have been reported, including the enhancement of β-cell functions or muscle endurance capacity, preservation of organelle functions, and improvement of the metabolic profiles [36–38]. Although this study only focused on the calcineurin activation for the activation mechanism of TFEB, further elucidation of the mechanism in response to hyperosmotic stress may provide a greater insight into drug development targeting the mTORC1-independent autophagy pathway for the treatment of kidney disease.

Chelating intracellular Ca^2+^ with BAPTA-AM prevented the hyperosmotic stress-induced TFEB nuclear translocation (Fig. 4b,c). The BAPTA-AM concentration of 50 μM sufficiently exceeded the threshold for the depletion of intracellular Ca^2+^ of NRK-52E cells used by the previous study [39]. TRPML1 channels localized in the lysosomes may partially account for this phenomenon, as the TRPML1 antagonist ML-SI3 significantly reduced the hyperosmotic mannitol-induced TFEB nuclear translocation (Fig. 6a,b). Although statistically insignificant, the ratio of TFEB nuclear translocation after the hyperosmotic stimulation remained slightly higher under the TRPML1 inhibition condition (0.89 ± 0.06) compared to the BAPTA-AM treatment condition (0.66 ± 0.03) (Fig. 4c, 6b). Because the main compartmentalized stores of Ca^2+^ in cells are the endoplasmic reticulum, mitochondria, and lysosomes [40], other Ca^2+^-releasing mechanisms may be involved in mediating or compensating for the increased intracellular Ca^2+^ level during the activation of calcineurin. In addition, the reduction in cell volume resulting from mannitol-induced cell shrinkage could be, in part, responsible for the increase in intracellular Ca^2+^ concentration. An understanding of the effects of hyperosmolarity on the other Ca^2+^ channels will help clarify the mechanism underlying changes in TFEB activation in proximal tubular epithelial cells.

ML-SI3, a chemical compound that acts as an antagonist of the TRPML family, abolished hyperosmotic-induced TFEB nuclear translocation (Fig. 6a,b). ML-SI3 is a potent inhibitor of TRPML1 (IC50: 1.6 μM) and TRPML2 (IC50: 2.3 μM), while it is a less effective inhibitor of TRPML3 (IC50: 12.5 μM) [41]. TRPML1 is ubiquitously expressed in mammalian cells and is mainly localized in the lysosomes, whereas TRPML2 and TRPML3 are expressed in specialized cells (e.g., immune cells and melanocytes) [15], emphasizing the importance of Ca^2+^ release through the activation of the TRPML1 channel. TRPML1 activation could partially account for the TFEB activation in response to hyperosmolarity, which is consistent with previous studies, demonstrating that TRPML1 contributes to autophagy by inducing the nuclear translocation of TFEB during starvation [42, 43]. Although there is still a great deal of uncertainty surrounding the mechanisms of TRPML1 activation by hyperosmotic stress, it is important to address the potential of the endogenous ligand of TRPML1, which can be activated by phosphatidylinositol 3,5-bisphosphate (PI(3,5)P2) [44]. PI(3,5)P2 is generated from PI(3)P through a PI5 kinase, and cells with PI(3,5)P2 deficiency exhibit enlarged endolysosomes and trafficking defects in endocytic pathways [45–47]. Thus, both TRPML1 and PI(3,5)P2 are involved in membrane-fusion processes such as lysosomal fusion with autophagosomes. Of interest, hyperosmotic stress reportedly increases PI(3,5)P2 levels more than 20-fold within a few minutes [44]. Considering ML-SI3 can negatively regulate the PI(3,5)P2 activation of TRPML1 [48], our findings indicate that ML-SI3 attenuates the hyperosmotic stress-induced autophagy, suggesting the involvement of calcium signaling via PI(3,5)P2/TRPML/TFEB in these responses. Although our study provides valuable insights through pharmacological approaches, further investigations are necessary to fully elucidate the underlying mechanisms, particularly through the use of knockdown techniques and complementary assays.

In conclusion, we showed that the hyperosmotic stress mediated by mannitol promotes TFEB’s nuclear translocation in tubular epithelial cells and identified TRPML1-induced activation of calcineurin as a crucial element in the mechanism underlying hyperosmotic stress-induced autophagy in these cells. These findings not only have implications for advancing our knowledge of the tubular epithelial cells’ response to hyperosmotic stress but may also contribute to providing insights into how cells translate mechanical stress into biological responses, specifically autophagy.

## Supplementary Information

Below is the link to the electronic supplementary material.

Supplementary file1 (DOCX 698 KB)
