# Supplementary material for: Hyperosmotic Stress Promotes the Nuclear Translocation of TFEB in Tubular Epithelial Cells Depending on Intracellular Ca2+ Signals via TRPML Channels
Source: Cell Mol Bioeng. 2025 Jan 21;18(1):39–52. doi: 10.1007/s12195-024-00839-6 (PMC11814421; doi:10.1007/s12195-024-00839-6)

**Suppl. Figure 1** Effect of hyperosmotic stress mediated by mannitol on TFEB expression in nuclear and cytoplasmic fractions. (a, b) Representative plots of TFEB, Lamin A/C, and GAPDH in the nuclear (left) and cytoplasmic (right) fractions of NRK-52E cells treated with 100 mM (a) and 200 mM (b) mannitol for 0, 0.5, and 1 h. (c, d) Quantitative analysis of TFEB expression levels from the Western blot images in (a) and (b), showing the relative nuclear-to-cytoplasmic TFEB expression normalized to 0 h. GAPDH was used as a cytoplasmic internal control, and Lamin A/C as a nuclear internal control. Data are presented as box and whisker plots with average (×), median, IQR, minimum value, and maximum value (100 mM: n = 3; 200 mM: n = 3). “n” represents the number of images analyzed. *P < 0.05 vs. 0-h treatment (Steel test). (e) Comparison of the TFEB nuclear/cytoplasmic ratio across different time points for 100 mM and 200 mM mannitol treatments, showing in (c) and (d). Summary data are presented as mean ± standard error (S.E.)


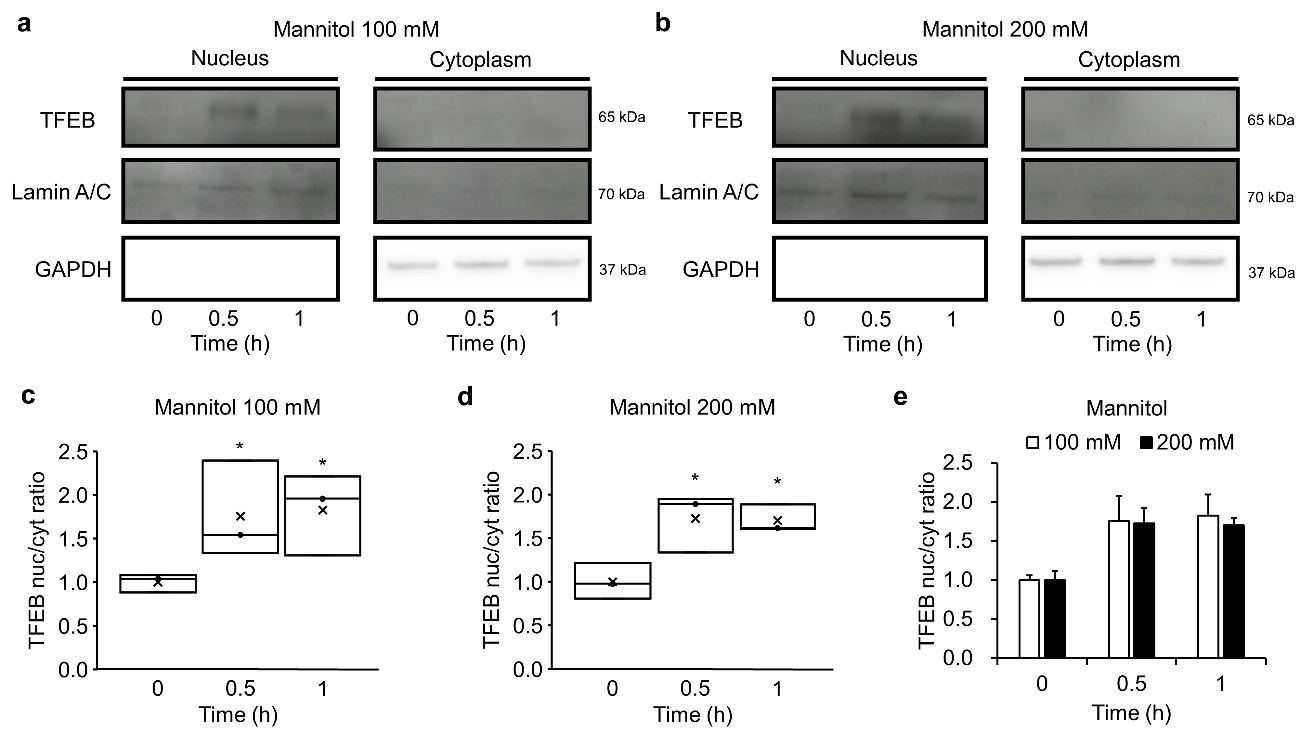


**Suppl. Figure 2** Effect of hyperosmotic stress mediated by mannitol and FK-506 on NFAT expression in nuclear and cytoplasmic fractions. (a) Representative plots of NFAT, Lamin A/C, and GAPDH in the nuclear (left) and cytoplasmic (right) fractions of NRK-52E cells treated with 200 mM mannitol for 0, 0.5, and 1 h. (b) Quantitative analysis of NFAT expression levels from the Western blot images in (a), showing the relative nuclear-to-cytoplasmic NFAT expression normalized to 0 h (c) Representative plots of NFAT, Lamin A/C, and GAPDH in the nuclear (left) and cytoplasmic (right) fractions of NRK-52E cells pretreated with 50 μM FK-506 for 15 min and subsequently treated with 200 mM Mannitol for 0, 0.5, and 1 h. (d) Quantitative analysis of NFAT expression levels from the Western blot images in (c), showing the relative nuclear-to-cytoplasmic NFAT expression normalized to 0 h. GAPDH was used as a cytoplasmic internal control, and Lamin A/C as a nuclear internal control. Data are presented as box and whisker plots with average (×), median, IQR, minimum value, and maximum value (n = 3). “n” represents the number of images analyzed. *P < 0.05 vs. 0-h treatment (Steel test).


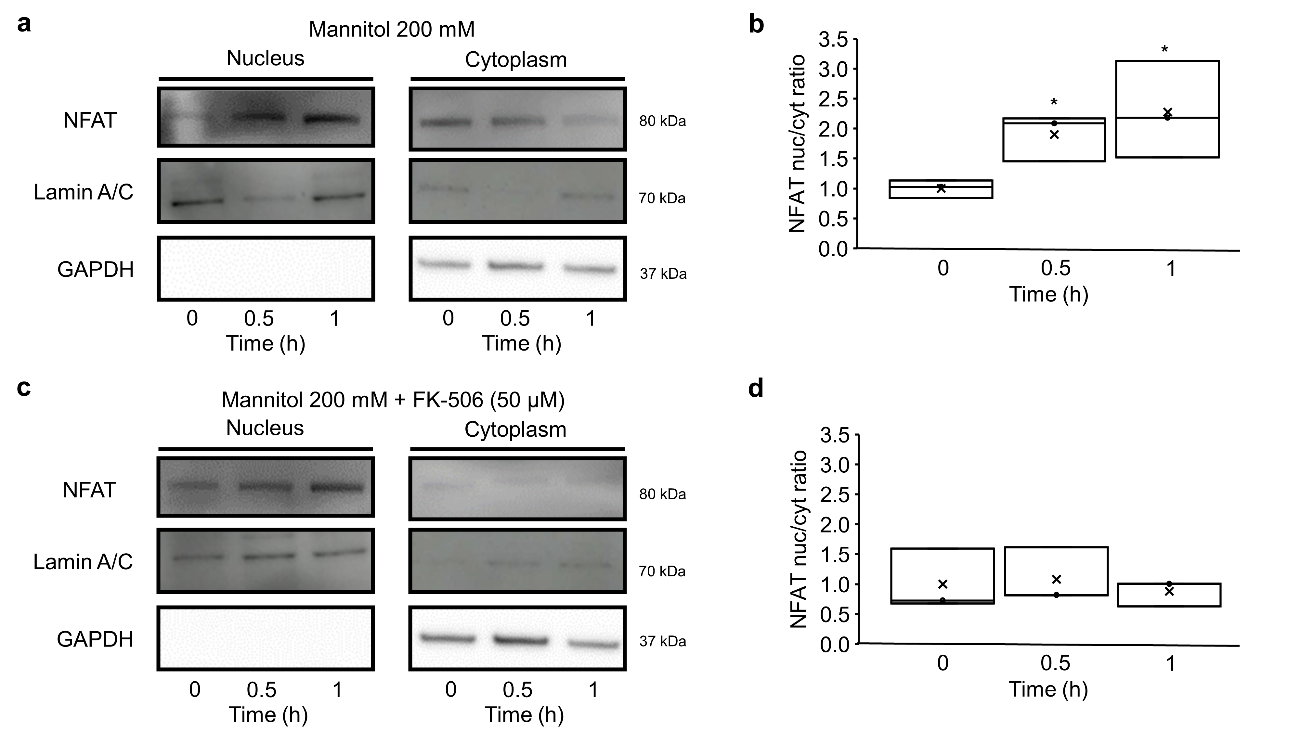


**Suppl. Figure 3** The effects of ML-SI3 on the protein expression of TFEB. Representative western blot images of TFEB in NRK-52E cells cotreated with mannitol (200 mM) and ML-SI3 (1 μM or 10 μM) for 1 h. GAPDH served as the loading control.


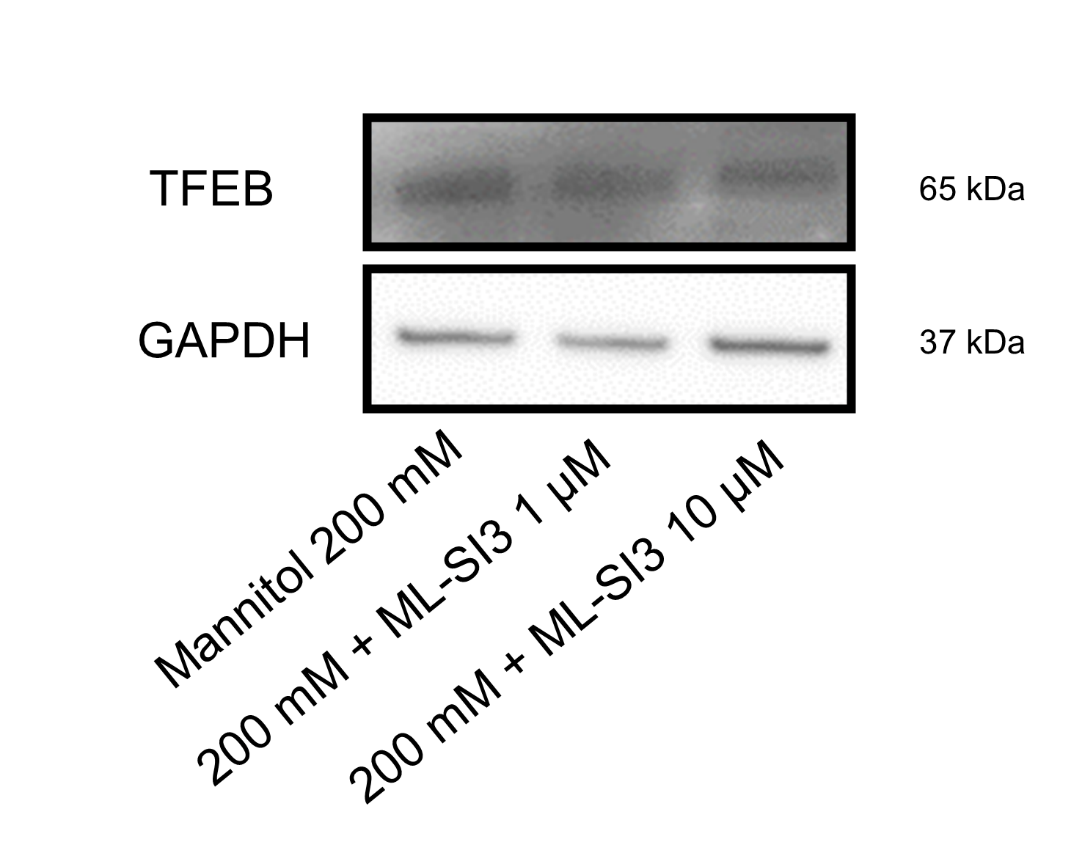

Supplement: Supplementary file 1 — Supplementary file1 (DOCX 698 KB) [file 12195_2024_839_MOESM1_ESM.docx]
